# From Static to Interactive: Transforming Data Visualization to Improve Transparency

**DOI:** 10.1371/journal.pbio.1002484

**Published:** 2016-06-22

**Authors:** Tracey L. Weissgerber, Vesna D. Garovic, Marko Savic, Stacey J. Winham, Natasa M. Milic

**Affiliations:** 1 Division of Nephrology and Hypertension, Mayo Clinic, Rochester, Minnesota, United States of America; 2 Department of Medical Statistics and Informatics, Medical Faculty, University of Belgrade, Belgrade, Serbia; 3 Division of Biomedical Statistics and Informatics, Mayo Clinic, Rochester, Minnesota, United States of America

## Abstract

Data presentation for scientific publications in small sample size studies has not changed substantially in decades. It relies on static figures and tables that may not provide sufficient information for critical evaluation, particularly of the results from small sample size studies. Interactive graphics have the potential to transform scientific publications from static reports of experiments into interactive datasets. We designed an interactive line graph that demonstrates how dynamic alternatives to static graphics for small sample size studies allow for additional exploration of empirical datasets. This simple, free, web-based tool (http://statistika.mfub.bg.ac.rs/interactive-graph/) demonstrates the overall concept and may promote widespread use of interactive graphics.

## Abstract 2

This article examines the potential for interactive graphics to transform scientific papers from static publications into interactive datasets and provides a web-based tool for creating interactive line graphs.

## Introduction

Scientific and technological advances have enhanced our ability to study the biology of health and disease. They have also changed the way that we access and share scientific information. Study preregistration websites, data repositories, reporting guidelines and recommendations, and checklists for statistical analysis are all designed to promote transparency and enhance the reproducibility of scientific results. Data presentation for scientific publications has not changed substantially, however, despite this growing emphasis on transparency and reproducibility. Scientists rely on static figures and tables that may not provide sufficient information for critical evaluation, particularly of the results from small sample size studies.

This paper aims to explore the potential of interactive graphics to transform scientific publications from static reports of an experiment into interactive datasets narrated by the authors. Small sample size studies offer excellent opportunities to explore interactive visualizations, as small datasets generally rely on a few key types of figures. These studies commonly use bar and line graphs that show summary statistics for continuous data and scatterplots that examine the relationship between two variables. Offering interactive alternatives to these static graphs may be a simple and effective strategy for promoting widespread use of interactive graphics. We have designed and present an interactive line graph as an alternative to the static graph for small sample size studies that allows for additional exploration of empirical datasets. In addition to demonstrating the overall concept, this simple, web-based tool may encourage utilization of interactive graphics and address growing demands to show individual-level data [1,2,3,4].

## Limitations of Traditional Line Graphs

**Fig 1:**
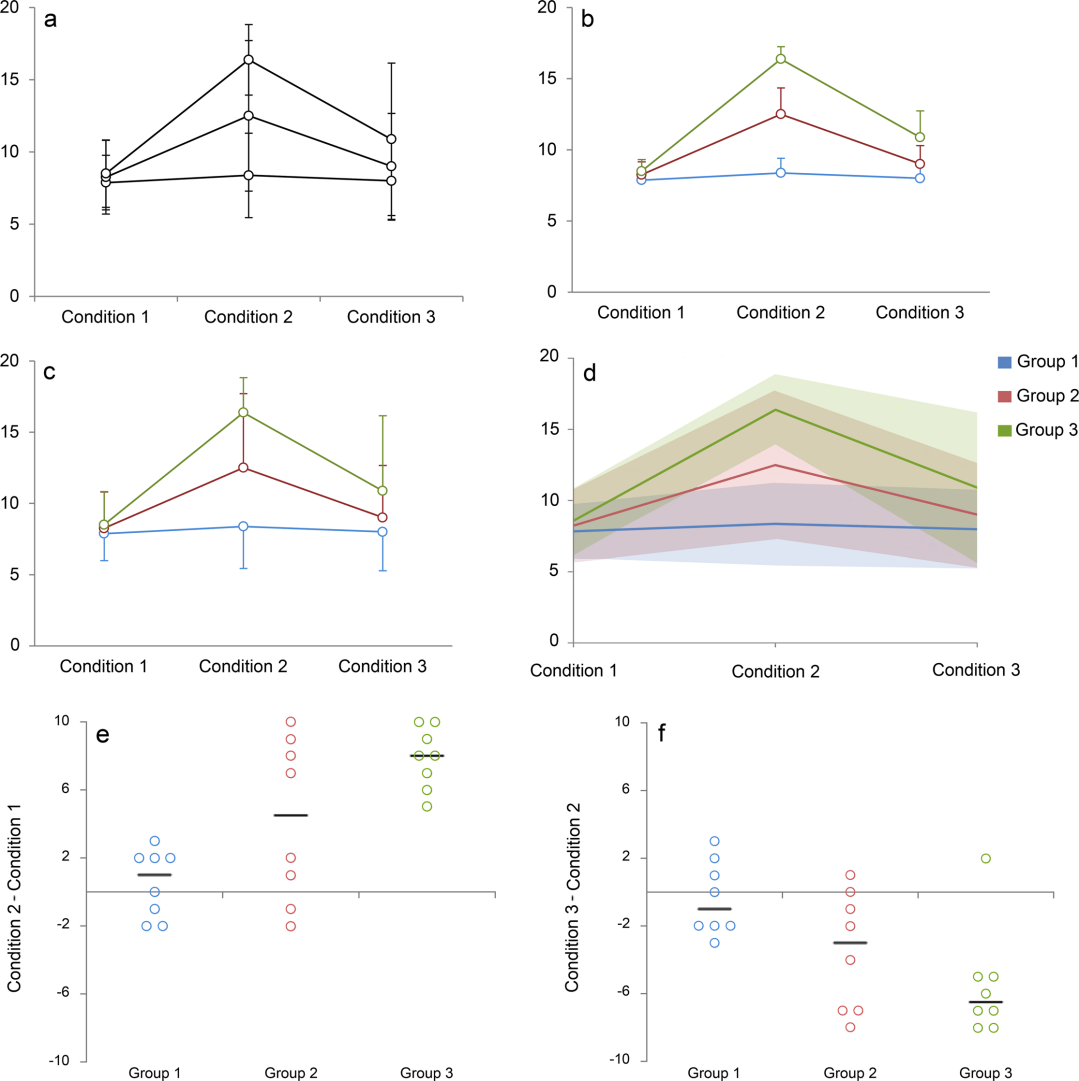
Reimagining the line graph. Panels A–C use traditional line graphs to present a simulated dataset as mean and standard error (Panel B) or mean and standard deviation (Panels A and C). While Panels A and C clearly indicate that there is overlap between groups, it difficult to assess the magnitude of the overlap. The error bars for Groups 2 and 3 overlap, while those for Group 1 go in the opposite direction. Panels D–F show selected figures that were created using our web-based tool for making interactive line graphs. Readers can view the interactive versions by uploading S1 Data into our web-based tool, then clicking on the name of each figure the “Graphs” heading. The lines in Panel D represent the group means, whereas the shaded regions represent one standard deviation above and one standard deviation below the mean. Replacing error bars (Panel C) with semitransparent shading (Panel D) makes it easier to identify regions where the groups overlap. The mean responses suggest that measurements for Group 1 do not change across the three conditions (Panel D). In contrast, Group 2 shows a small response to Condition 2, whereas Group 3 shows a larger response. However, examining individual-level data showing changes from Condition 1 to Condition 2 (Panel E) reveals that Group 2 includes responders and nonresponders. Response patterns for the responders are similar to the responses observed among individuals in Group 3, whereas response patterns for the nonresponders are similar to those of individuals in Group 1. Panel F shows that while values for most individuals in Group 3 decreased between Conditions 2 and 3, one individual experienced a slight increase. This observation is a clear outlier. The lines for Panels E and F represent the median change.

A recent systematic review of original research articles published in top physiology journals demonstrated that 61% of papers contain at least one line graph, making this the second most common type of figure used to present continuous data [1]. Line graphs are designed for longitudinal data; lines are used to show that measurements were repeated on the same participant, specimen, or sample. Measurements are typically performed at predetermined sets of time points or conditions in experimental studies. The lines estimate the pattern of response by assuming a linear change between each consecutive set of time points or experimental conditions. This is fundamentally different from regression and other types of analysis, in which lines are used to illustrate trends that were estimated using one measurement per participant, specimen, or sample. Line graphs focus on how differences between the means for each group change across time points or conditions. However, they do not provide two important pieces of information. First is the amount of overlap between different groups, as less overlap indicates that the difference is more important. Second is information as to whether all individuals in the same group follow a consistent response pattern. This information is difficult or impossible to obtain using the standard line graph. The degree of overlap between groups is typically illustrated by showing error bars that represent the standard deviation. However, error bars for different groups frequently overlap (Fig 1, Panel A), making it difficult to determine where the error bars for each group end. Several strategies are used to address this problem. The most common approach is to use error bars to show the standard error (Fig 1, Panel B), which is smaller than standard deviation. This reduces the likelihood that error bars for different groups will overlap; however, standard errors measure the precision of the mean rather than the variability in the sample. An alternate approach is to use unidirectional error bars, which are oriented away from other groups (Fig 1, Panel C). In this case, it is difficult to estimate the position of the missing error bars to assess the amount of overlap between groups. Another option is to stagger the position of overlapping data points on the x-axis; however, few graphical packages offer this alternative.

**Fig 2:**
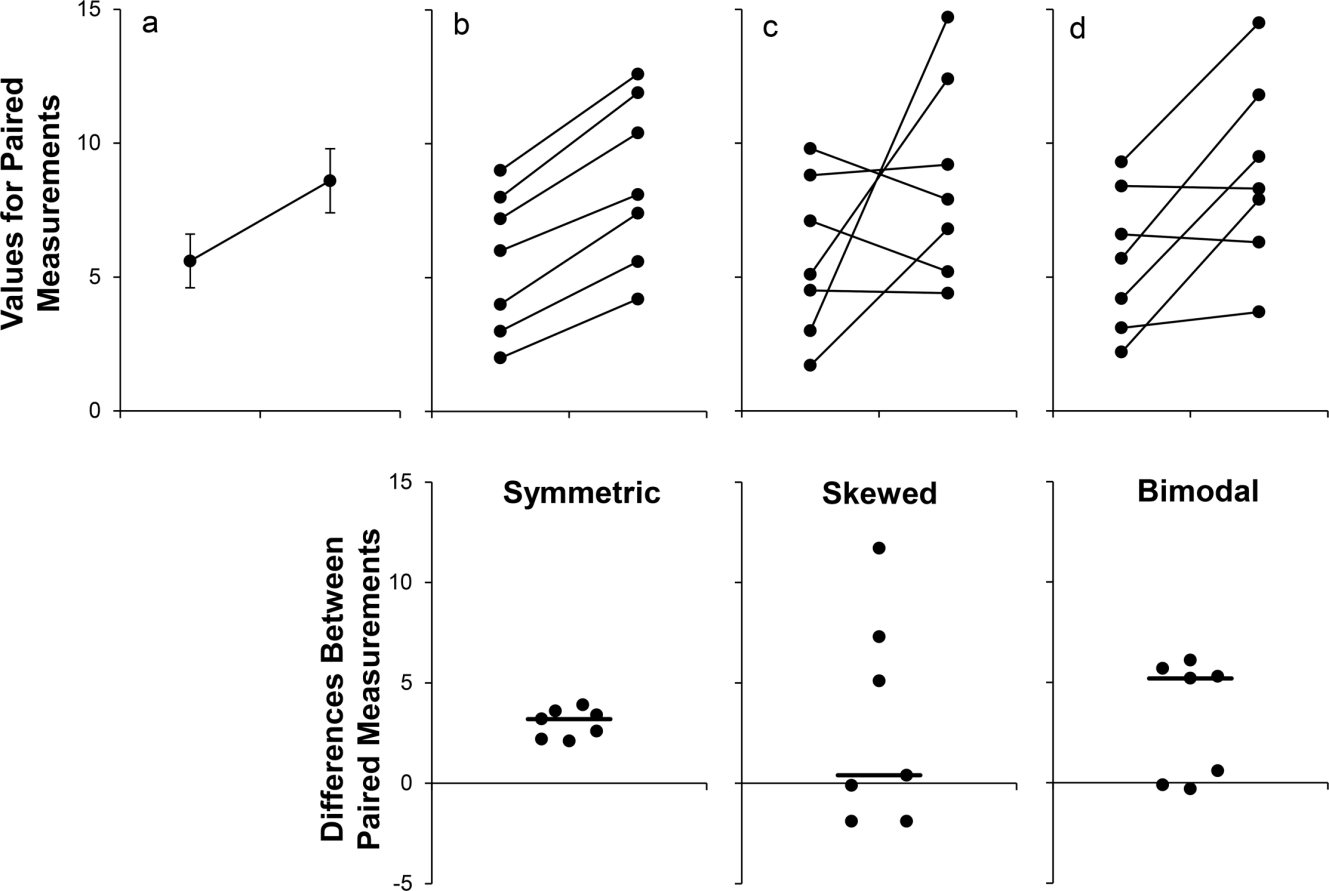
Many different datasets can lead to the same line graph. The line graph (mean ± standard error) provides no information about whether changes are consistent across individuals (Panel A). The scatterplots shown in the Panels B–D reveal very different patterns of change, even though the means and standard errors differ by less than 0.3 units. The lower scatterplots showing the differences between measurements allow readers to quickly assess the direction, magnitude, and distribution of the changes. The solid lines show the median difference. In Panel B, values for every subject are higher in the second condition. In Panel C, there are no consistent differences between the two conditions. Panel D suggests that there may be distinct subgroups of “responders” and “nonresponders.” Adapted from Weissgerber et al. [1].

The common practice of displaying summary statistics can be misleading, as many different data distributions can lead to the same graph (Fig 2) [1]. The actual data may suggest different conclusions from the summary statistics. This problem is accentuated by the small sample sizes often used in basic science research. In 75% of papers published in top physiology journals, the smallest group shown in a figure had six independent observations or fewer, whereas the largest group shown in a figure had 15 independent observations or fewer [1]. A recent study reported that eight animals per group is a typical sample size for preclinical research [5]. Outliers are common in such small datasets, and it is difficult to determine the distribution of the data. This is problematic, as standard line graphs do not show values for individual participants. The sample size for each group cannot be determined, nor can the viewer assess whether response patterns are similar for all individuals in a particular group.

## Current Alternatives to Traditional Line Graphs

While several alternatives to the line graph have been proposed [6,7], the existing options have important limitations. Templates for creating graphics for paired or matched data were provided in our previous paper [1]. The templates create univariate scatterplots showing differences for each individual as well as “spaghetti plots” in which lines are used to connect paired values (as shown in Fig 2, upper graphs of Panels B, C, and D). This approach does not scale well for larger datasets or for small datasets with more than two time points or conditions. Showing one line for each individual often leads to a complicated and uninformative graphic with many crossing lines. It may also be difficult to distinguish among individuals in different groups, especially when groups overlap. The reliance on black and white figures in scientific papers exacerbates these problems. A variety of other strategies have been proposed, including small multiples [6] and lasagna plots [7]. S1 Text briefly outlines several options and provides examples and references. Many of these strategies are most effective for datasets without groups, in which each line represents an observation of interest. Other static alternatives to the traditional line graph make it difficult to determine whether responses are consistent among all individuals within a particular group. Strategies such as the lasagna plot provide individual level data; however, the lasagna plot was designed for large datasets and is less effective in small studies.

## An Interactive Alternative to Traditional Line Graphs

Interactive line graphs may provide additional information needed to interpret longitudinal data in small studies. We developed a simple, free, web-based tool (http://statistika.mfub.bg.ac.rs/interactive-graph/) that allows users to quickly create interactive line graphs for small datasets. These graphs have four key features, allowing for rapid examination of different aspects of the data (Box 1):

1. View different summary statistics: the base graph shows the central tendency and variation in each group for each condition or time point. The user can adjust the graph to view the mean, mean and standard deviation, mean and standard error, mean and 95% confidence interval, median, median and interquartile range, or median and range. Measures of variation for each group are shown as a semitransparent shaded region, allowing one to assess the magnitude of the overlap among observations from different groups.
2. Display lines for some or all individuals in each group: the line for each participant or sample in the dataset can be turned on or off individually, allowing one to view any subset of individuals in the dataset.
3. View a subset of groups, conditions, or time points: these options allow the viewer to focus on a subset of groups, conditions, or time points.
4. View change scores for any two conditions or time points: the “Difference Plot” tab displays a univariate scatterplot that shows change scores for each individual in the dataset. This allows for comparisons of the magnitude, direction, and consistency of changes across groups.

**Box 1. Data Exploration Using the Interactive Line Graph**

Interactive line graphs can be quickly created using a web-based application that does not require any programming expertise or specialized skills—users simply enter or upload data and customize the graph axes and labels. The insight gained from an interactive line graph will depend on the empirical dataset. In addition to enhancing readers' understanding of the data, the interactive line graph may help authors to select static graphs that most effectively illustrate key findings for print publication. A simulated dataset is provided to illustrate these points (S1 Data). The interactive line graph can be viewed by uploading this simulated dataset into the web-based tool (http://statistika.mfub.bg.ac.rs/interactive-graph/upload). Fig 1 shows traditional line graphs for this dataset (Panels A–C), followed by selected static graphs that were created using the web-based tool (Panels D–F). The traditional line graphs showing mean ± standard error (Panel B) and mean ± standard deviation (Panels A and C) provide no information about individual responses and make it difficult to assess the degree of overlap between groups. When the mean ± standard deviation graph is recreated using our web-based tool (Panel D), the overlapping and unidirectional error bars are replaced by semitransparent shaded regions. Differences in shading make it easier to identify regions where the standard deviations for different groups overlap. The average values suggest that there is no response in Group 1, an intermediate response in Group 2, and a large response in Group 3. However, the individual change scores examining the differences between Conditions 1 and 2 tell a different story (Panel E). Group 2 seems to include “responders” and “nonresponders.” Nonresponders follow the same pattern of change as individuals in Group 1, whereas the magnitude of change in responders is similar to the responses observed among individuals in Group 3. Averaging these two subgroups gives the misleading impression of an intermediate response in Group 2.

The tool allows for both (1) the integration of static graphics into a publication as a .tiff file and (2) downloading of a data file for a customized interactive graphic, which can be presented in the paper supplement. As color coding is used to present different groups, the tool includes a color blind working mode. All interactive line graph features can be viewed in a color blind–safe color scheme. A black-and-white mode is also included for less complex graphs.

## From Static to Interactive Scientific Publishing

A recent editorial highlighted the static nature of data presentation as a major limitation of scientific publications [8]. There are several potential benefits to making interactive graphics common features of publications for small sample size studies. Interactive graphics can provide crucial information that cannot be obtained from a static graphic. They may be valuable tools for promoting transparency, reproducibility, and open science in an era when these factors are increasingly valued [9,10,11]. Customized interactive graphics have already been presented by journals [12] and authors [13,14] to complement research articles. Anecdotal reports suggest that this can be an effective strategy for increasing interest in published research [13]. Interactive data visualizations could fundamentally change the way authors, reviewers, and readers understand and interpret research data. However, the application of interactive graphics in scientific publications will be dependent on both author and journal acceptance. Author-level solutions, such as the interactive line graph described in this paper, would allow authors to create interactive graphics for individual papers and include them in the data supplement. Journal-level solutions would allow journals to include interactive graphics in the web versions of all papers published in the journal.

## Conclusions

This paper presents a "proof of concept" example that demonstrates how interactive alternatives to static graphics for small sample size studies allow for additional exploration of empirical datasets and illustrates the types of tools that are needed to promote widespread use of interactive graphics. The principles described above can be applied to other types of figures and tables, including those applicable to big datasets. Most scientists use electronic devices to access scientific publications, yet the interactive potential of these technologies remains untapped. Exploring more dynamic alternatives is crucial as we enter an era of transparent and open science.

## Supporting Information

S1 Data Example of an interactive line graph. This example can be viewed by uploading S1 Data into the web-based tool (http://statistika.mfub.bg.ac.rs/interactive-graph/).
(XML)

S1 Fig Small multiples. Panel A: Data for each individual in Group 2 from the dataset presented in Fig 1 are shown as small multiples [6]. Panel B: Horizontally aligned small multiples suggest that the peak response occurs earlier among individuals in the first group (red lines), compared to individuals in the second group (blue lines). Panel C: Select individuals from Group 1 and Group 3 of the dataset presented in Fig 1 are shown as small multiples. The shaded region shows one standard deviation above the mean and one standard deviation below the mean for Group 1 (blue) and Group 3 (green) of the dataset shown in Fig 1. Each line represents the response for one individual.
(TIF)

S2 Fig Changes from baseline [15]. The response for each individual is presented as the change from the baseline value. Horizontal small multiples are used to highlight differences in the magnitude of the response among individuals.
(TIF)

S3 Fig Spaghetti plots. Panel A: Individual responses for the dataset shown in Fig 1 are presented in a spaghetti plot. Panel B: Responses for individuals in Group 2 of the dataset shown in Fig 1 are presented in a spaghetti plot. Panel C: The spaghetti plot shown in Panel B is divided into a series of small multiples. Each graph highlights the response of a different individual in the dataset [16].
(TIF)

S4 Fig Showing responses for selected individuals [17]. Changes in placental growth factor were examined longitudinally in women who had normotensive pregnancies (*n* = 24) and women who developed preeclampsia (*n* = 15). The points show observations from all women in the dataset (mode = 3 measurements per woman; range 1–4 measurements per woman). Lines show the pattern of change for one individual in each tertile in both the normotensive pregnancy and preeclampsia groups.
(TIF)

S1 Text Static alternatives to the line graph. (DOCX)
