# Correction: From Static to Interactive: Transforming Data Visualization to Improve Transparency

**DOI:** 10.1371/journal.pbio.1002545

**Published:** 2016-08-17

**Authors:** Tracey L. Weissgerber, Vesna D. Garovic, Marko Savic, Stacey J. Winham, Natasa M. Milic

The original S1 Data file was corrupted due to missing characters at the end of the file. Please download this corrected version to use in the web-based tool provided below.

## Supporting Information

S1 Data Example of an interactive line graph. This example can be viewed by uploading S1 Data into the web-based tool (http://statistika.mfub.bg.ac.rs/interactive-graph/).
(XML)
