# Supplementary figures and images for: From Static to Interactive: Transforming Data Visualization to Improve Transparency
Source: PLoS Biol. 2016 Jun 22;14(6):e1002484. doi: 10.1371/journal.pbio.1002484 (PMC4917243; doi:10.1371/journal.pbio.1002484)

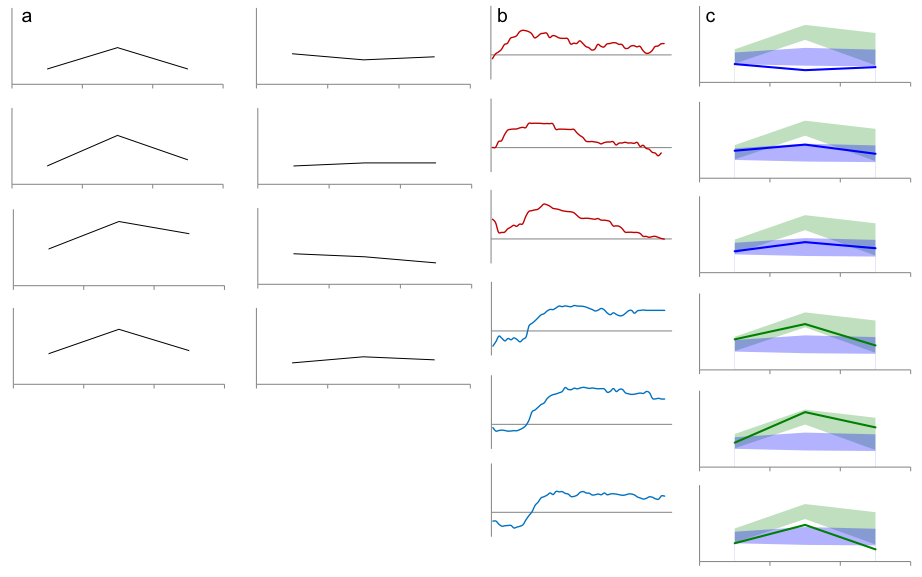

Supplement: S1 Fig — Panel A: Data for each individual in Group 2 from the dataset presented in Fig 1 are shown as small multiples [6]. Panel B: Horizontally aligned small multiples suggest that the peak response occurs earlier among individuals in the first group (red lines), compared to individuals in the second group (blue lines). Panel C: Select individuals from Group 1 and Group 3 of the dataset presented in Fig 1 are shown as small multiples. The shaded region shows one standard deviation above the mean and one standard deviation below the mean for Group 1 (blue) and Group 3 (green) of the dataset shown in Fig 1. Each line represents the response for one individual. (TIF) [file pbio.1002484.s002.tif]

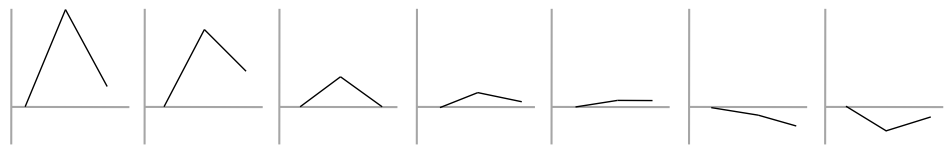

Supplement: S2 Fig — The response for each individual is presented as the change from the baseline value. Horizontal small multiples are used to highlight differences in the magnitude of the response among individuals. (TIF) [file pbio.1002484.s003.tif]

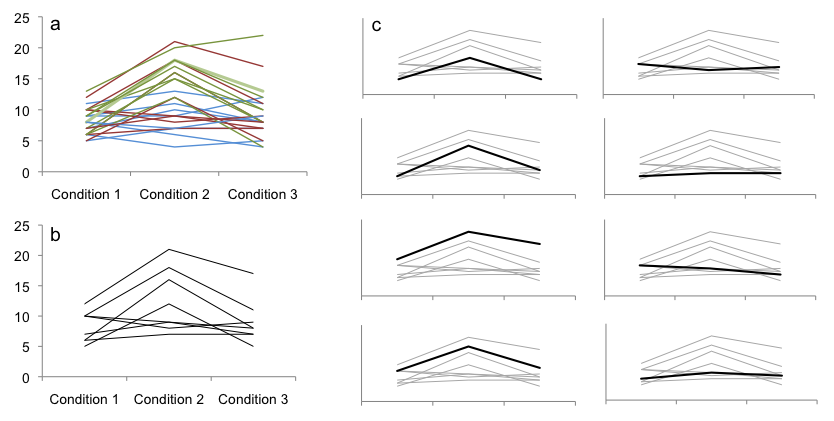

Supplement: S3 Fig — Panel A: Individual responses for the dataset shown in Fig 1 are presented in a spaghetti plot. Panel B: Responses for individuals in Group 2 of the dataset shown in Fig 1 are presented in a spaghetti plot. Panel C: The spaghetti plot shown in Panel B is divided into a series of small multiples. Each graph highlights the response of a different individual in the dataset [16]. (TIF) [file pbio.1002484.s004.tif]

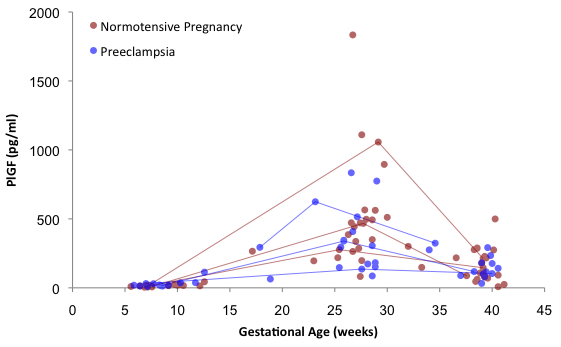

Supplement: S4 Fig — Changes in placental growth factor were examined longitudinally in women who had normotensive pregnancies (n = 24) and women who developed preeclampsia (n = 15). The points show observations from all women in the dataset (mode = 3 measurements per woman; range 1–4 measurements per woman). Lines show the pattern of change for one individual in each tertile in both the normotensive pregnancy and preeclampsia groups. (TIF) [file pbio.1002484.s005.tif]
