# Supplementary material for: From Static to Interactive: Transforming Data Visualization to Improve Transparency
Source: PLoS Biol. 2016 Jun 22;14(6):e1002484. doi: 10.1371/journal.pbio.1002484 (PMC4917243; doi:10.1371/journal.pbio.1002484)
Supplement: S1 Text — (DOCX) [file pbio.1002484.s006.docx]

**Static Alternatives to the Line Graph**

This section briefly outlines several alternatives to the line graph and provides examples and references for readers who would like more information. Many of these strategies are most effective for datasets without groups, where each line represents an observation of interest (i.e. comparing changes in child mortality for different countries, where each line represents one country). Some strategies are designed for large datasets and are less effective in small sample size studies.

**Small Multiples:** Small multiples can be an effective strategy for drawing the viewers’ attention to differences in the time course of change, or differences in the magnitude of change. Unlike interactive graphics, these graphs can be included in printed versions of the manuscript. Small multiples refer to a series of small graphs with the same scales on the x and y-axes (S1 Fig, Panel a) [[1](#_ENREF_1)]. Each graph shows a single line for one participant in the dataset [[1](#_ENREF_1)]. When working with very small datasets, small multiples may be aligned either vertically or horizontally. Larger datasets may combine both approaches, creating a grid of small graphs. The major advantage of small multiples is that they show data for all participants in a format that allows readers to quickly compare individual observations. A disadvantage is that one or both axes are often compressed, which can make it difficult to distinguish between lines for individual participants. Axis compression is particularly problematic when the dataset is strongly skewed or includes outliers. Vertical alignment of small multiples often compresses the y-axis, however this approach can be extremely useful for illustrating differences in the time course of change (x-axis). In panel b of S1 Fig, quickly scanning the column of small multiples reveals that values for Group 1 participants (red lines) peak faster and return to baseline sooner, when compared to response curves from Group 2 participants (blue lines). In contrast, horizontal alignment of small multiples often compresses the x-axis. This approach can be very useful in detecting differences in the magnitude of change.

Although small multiples can be very effective in the situations described above, they may be most useful in situations where there are no groups and each line represents an observation of interest. For example, small multiples would be effective in comparing changes in the incidence of malaria over time for different countries, where each line represents one country. When a figure includes several different groups with multiple observations per group, axis compression can make it difficult to determine how groups differ. One possible solution is to show summary statistics for each group in the background of each graph (S1 Fig, Panel c). However, the added complexity can make this simple graphic difficult to interpret.

**Using trend lines to show changes from baseline:** The designer creates a series of small multiples, in which the y-axis shows the change from baseline (S2 Fig) [[2](#_ENREF_2)]. Starting all graphs at the same value (i.e. no change from baseline) allows viewers to quickly compare the magnitude and direction of change for different individuals. This technique is most useful for datasets in which the baseline value is not important.

**The spaghetti plot:** Each individual in the dataset is represented by a single line (S3 Fig, Panels a and b). Spaghetti plots work best when lines for different individuals rarely cross. This is most likely for datasets with a small number of participants and time points or conditions. Spaghetti plots become increasingly complicated and uninformative as the number of participants and time points or conditions increases. When the graph is too complex, the reader can no longer visually distinguish data from individual participants.

In very large datasets with many crossing lines, spaghetti plots can be used to create shading effects where values that are more common appear darker than values that are less common. Designers can create these effects by using thin, semi-transparent lines of the same color to graph data for all individuals in a particular group. This strategy is valuable for showing the distribution of the data and degree of overlap between groups, however the viewer will not be able to examine response patterns of individual participants.

**Deconstructing the spaghetti plot by emphasizing one line at a time:** The designer creates a series of small multiples, each of which emphasizes one individual in the dataset (S3 Fig, Panel c) [[3](#_ENREF_3)]. The line for the individual that is emphasized may be thicker or colored, whereas lines for the remaining individuals are de-emphasized (i.e. thinner, gray). As with small multiples, this approach is most effective in situations where there are no groups and each line represents an observation of interest.

**Show all data points; then use lines to display individual responses for a few participants:** The designer creates a scatterplot showing all data points for all individuals in the dataset; then uses lines to show individual responses for a small number of participants (S4 Fig) [[4](#_ENREF_4)]. This subset may be selected at random, or chosen based on a mathematical characteristic (i.e. one participant in each quartile), pre-defined groups (i.e. trained vs. sedentary) or groups based on response patterns (i.e. responders vs. non-responders). This approach works best when the response patterns are similar for most individuals in the groups that are shown. The graph may be misleading if responses patterns are highly variable and responses for the subset of individuals that are shown are not representative. Furthermore, this type of graphic is most effective when the x-axis is a continuous variable and values differ among participants. When all participants are tested at the same time points, the graph becomes a series of strip plots with overlapping values. Using semi-transparent data points can help to the viewer to identify values with overlapping observations.

**Lasagna plots:** Changes of different magnitudes are represented by different colors or different shades of the same color [[5](#_ENREF_5)]. For example, a large decrease may be represented by dark blue, a small decrease may be represented by light blue, a small increase may be represented by light red, and a large increase may be represented by dark red. The designer then creates a grid where each individual is represented by a different row, and each time point or condition is represented by a different column. Colors representing changes for each individual at each time point or condition are entered into the grid. The rows are then re-arranged so that individuals with a similar response patterns (i.e. colors) are grouped together. While lasagna plots allow the viewer to quickly identify subgroups of individuals with distinct response patterns, the use of arbitrary color scales can make it difficult to estimate the magnitude of the changes. These figures are most useful for showing whether values increase or decrease, rather than by how much. In datasets with very small sample sizes, the authors may not have enough data to identify subgroups of participants with different response patterns.

**References**

1. Tufte E (1983) The visual display of quantitative information. Cheshire, Connecticut: Graphics Press.

2. Bella On a Par, Bella Consultants, March 30, 2012, <http://www.bella-consults.com/page/4>.

3. Knaflic CN Strategies for avoiding the spaghetti graph, Storytelling with Data, March 14, 2013, <http://www.storytellingwithdata.com/blog/2013/03/avoiding-spaghetti-graph>.

4. Diggle PJ, Heagerty PJ, Liang KY, Zeger SL (2002) Analysis of Longitudinal Data. Oxford: Clarendon Press.

5. Swihart BJ, Caffo B, James BD, Strand M, Schwartz BS, Punjabi NM Lasagna plots: a saucy alternative to spaghetti plots. Epidemiology. 2010;21: 621-625.
